# Dendritic cells that phagocytose apoptotic macrophages loaded with mycobacterial antigens activate CD8 T cells via cross-presentation

**DOI:** 10.1371/journal.pone.0182126

**Published:** 2017-08-02

**Authors:** Patricia Espinosa-Cueto, Alejandro Magallanes-Puebla, Carlos Castellanos, Raul Mancilla

**Affiliations:** Departamento de Inmunología, Instituto de Investigaciones Biomédicas, Universidad Nacional Autónoma de México, Mexico City, Mexico; Rutgers University, UNITED STATES

## Abstract

While homeostatic apoptosis is immunologically silent, macrophage apoptosis during *Mycobacterium tuberculosis* infection can potentially induce an immune response against the mycobacteria. To examine the role of dendritic cells in this response, macrophage apoptosis was induced by incubating the macrophage with cell wall extracts of mycobacteria expressing LpqH. The apoptogenic proteins of the cell wall extracts were engulfed by the macrophage and then were translocated from the cytosol to the nuclei of the dying cells. Dendritic cells that engulfed the apoptotic macrophages acquired an immunogenic phenotype that included upregulation of MHC-I, increased expression of the costimulatory molecules, CD40, CD80, and CD86, and increased production of IL-12, IL-10, TNF-α, and TGF-β. In addition, the dendritic cells triggered a proliferative response of CD8^+^ T cells with IFN-γ production via cross-presentation. Taken together, these findings support a model in which phagocytosis of whole apoptotic cells carrying mycobacterial antigens promotes a potentially protective immune response.

## Introduction

An immune response against *Mycobacterium tuberculosis* (Mtb) involves both innate and adaptive immune mechanisms [1]. Dendritic cells (DCs) that are located near the alveoli can capture incoming bacilli, and then travel to the hilar lymph nodes to process and present antigens to T cells [2]. Thus, an adaptive immune T cell response is initiated and mediated by CD4 and CD8 cells [1]. A number of observations support the view that apoptosis of macrophages (MØ) infected by Mtb constitutes an innate immune response [3] Currently, there is much interest in this response since it has been well established that homeostatic apoptosis regulates tissue turnover in the body to maintain stable cell populations, yet this process is not immunogenic [4]. Moreover, the uptake of apoptotic cells by DCs and MØs may lead to immunologic tolerance with increased production of anti-inflammatory cytokines, such as IL-10 and TGF-β, and downregulation of major histocompatibility complexes (MHCs) and costimulatory molecules [4, 5]. Nonetheless, it is now well recognized that apoptosis can be also immunostimulatory. For example, molecules with immunogenic properties have been detected in apoptotic cells, including heat shock proteins, tumor antigens, cell surface-exposed calreticulin, and HMGB1 [6, 7]. It has also been reported that apoptotic cells carrying antigens evoke an immune response [8, 9].

*In vitro* studies have shown that Mtb-infected MØs may clear an infection or die [3]. Initially, it was demonstrated that MØ death exhibited features of apoptosis, including exposure of phosphatidyl serine and breaks in nuclear DNA [10]. More recently, it has been shown that Mtb-infected MØs may undergo necrosis, particularly when they are infected with virulent strains. This was characterized by cell swelling and plasma membrane rupture which facilitated the release of bacilli and dissemination of infection [3]. The concept that apoptosis of Mtb-infected MØ is a form of altruistic suicide is mainly based on *in vitro* observations. In particular, a reduction in mycobacteria viability [11, 12] and the capacity of virulent strains to inhibit apoptosis by upregulating antiapoptotic molecules of the BCL-2 family have been observed [13]. Correspondingly, antiapoptotic genes have been identified in virulent Mtb strains, and their deletion resulted in a more efficient immune response [14]. Individuals with active TB express genes that are associated with the extrinsic apoptosis pathway, including TNF-α, Fas, FasL, and caspase 8 [15]. More recently, it has been observed that DCs engulf membrane-bound microvesicles that have been released from mycobacteria-infected MØs in early apoptosis [16]. These microvesicles are also referred to as blebs. DCs that engulf apoptotic blebs acquire the ability to trigger an immune response of T cells. It is unknown if the apoptotic bodies that remain after the release of blebs are immunogenic [17]. This is relevant because it has been reported that blebs and apoptotic bodies differ in their effects on immunity in order to gain a better understanding of the role played by host cell apoptosis in antimycobacterial immunity, we studied the maturation profile, autocrine cytokine production and the T cell response elicited by bone marrow-derived DCs that phagocytosed whole apoptotic MØs carrying mycobacterial proteins [18].

## Materials and methods

### Ethics statement

The use of animals and the experimental procedures for this study were approved by the Comisión Institucional para el Cuidado y Uso de Animales del Laboratorio (CICUAL), Bioethics Committee of the Instituto de Investigaciones Biomédicas (Universidad Nacional Autónoma de México) according to established protocols.

### Reagents and antibodies

The following antibodies and reagents were obtained from BioLegend (San Diego, CA. USA): Annexin V (FITC); CD11c (PerCP/Cy5.5, clone N418); MHC-I (FITC, clone SF1-1.1); MHC-II (PE, clone M5/114.15.2); CD40 (APC, clone 3/23); CD80 (Alexa Fluor 488, clone 16-10A1); CD86 (APC, clonePO3); DC90.2,Thy (PE, clone 30- H12); CD4 (PE/Cy7, clone GK1.5); CD8a (APC, 53–6.7); CD16/32, FC blocker (clone 93), and ELISA MAX standard set for mouse IFN-γ, IL-12/IL-23 (P40), IL-10, and TNF-α. Additional reagents were purchased as indicated: an Annexin V MicroBead Kit (Miltenyi Biotec, Bergisch Gladbach, Germany), the general caspase inhibitor Z-VAD-FMK (BD Pharmigen, San Diego, CA, USA). ProLong Gold anti-fade reagent with DAPI, 5-(and-6)-carboxyfluorescein diacetate (CFSE), 7-aminoactinomycin D (7-AAD) and succinimidyl ester (5(6)-CFDA (Invitrogen Molecular Probes, Eugene, OR, USA), the lipophilic membrane markers PKH26-GL and PKH67-GL, lipopolysaccharide (LPS), 3,3'-diaminobenzidine (DAB), bafilomicyn A1, and saponin (Sigma Aldrich, St. Louis, MO, USA), an *in situ* Cell Death Detection Kit and TMR red (Roche Applied Science, Indianapolis, IN, USA), recombinant murine granulocyte-macrophage colony-stimulating factor (GM-CSF) (PeproTech, Rocky Hill, NJ, USA), antibodies recognizing histone H2B and histone H4 (Santa Cruz Biotechnology, Palo Alto, CA, USA), and MG-132 (Calbiochem Millipore, Billerica, MA, USA).

### Mycobacteria cultures and isolation of cell wall proteins

A recombinant *M*. *smegmatis* strain was generated by electroporation with the plasmid p16R1-19, which has an additional 1.8 kb fragment including the *M*. *tuberculosis* LpqH gene (kindly donated by Y. Zhang, MRC Tuberculosis and Related Infections Unit, Hammersmith Hospital, London, UK). The *M*. *smegmatis* expressing LpqH (MsmegLpqH) and its wild-type counterpart were grown for 5–7 d in Middlebrook 7H9 medium supplemented with 2% glucose and hygromycin B (50 μg/ml). Expression of LpqH in the cell wall of the transformed strain was verified by immunoblot with the monoclonal antibody (mAb) IT-19 (kindly donated by TB Vaccines Testing and Research Materials Contract, Colorado State University, CO, USA). MsmegLpqH and twild-type *M*. *smegmatis* was sonicated at 60 KHz on ice (20 cycles, 1 min each) to obtain a cell wall extract.

### Culture of bone marrow-derived MØ and induction of apoptosis

Bone marrow-derived MØs were obtained from 6/7-weeks-old Balb/cJ mice. Briefly, the mice were euthanized in CO_2_ chamber and the femurs and tibiae were collected. These bones were flushed with RPMI 1640 medium to extrude bone marrow into a cell culture dish that contained RPMI 1640 medium. The bone marrow cells were subsequently passed through a 100-mm mesh and red cells were depleted with ACK lysis buffer. The cells were rinsed, and then resuspended in culture medium composed of RPMI-1640, 20% heat-inactivated fetal bovine serum (FBS), 1% non-essential aminoacids, 1% antibiotic-antimycotic, and 1% sodium pyruvate. The cells were grown at 37°C with 5% CO_2_. To induce apoptosis, after 10 d of culture MØs were incubated on ice with EDTA 0.5mM and detached with a scraper. Cell viability of 95% was attained. To assess MØs purity, flow cytometry with a mAb to F4/80 was carried out. To induce apoptosis 5 x 10^5^ MØs were incubated for 24 h with 50 μg MsmegLpqH cell wall extract protein. Apoptosis was also induced by exposing MØs to ultraviolet (UV) light for 1 h. MØs were detached from the plates with a scraper and cytospin slides were subjected to terminal deoxynucleotidyl transferase dUTP nick end labeling (TUNEL) to detect DNA breaks. The slides were mounted with ProLong Gold anti-fade reagent and DAPI to label cell nuclei for fluorescence microscopy. To assess apoptosis by flow cytometry, MØs were rinsed with binding buffer (0.1 M Hepes (pH 7.4), 1.4 M NaCl, 25 mM CaCl_2_) and then stained with Annexin-V and 7-AAD. After 15 min, the cells were examined with a FACSCalibur Flow Cytometer (Becton-Dickinson, Mountain View, CA, USA). To isolate apoptotic MØs, the cells were centrifuged 5 min at 453 x g and the supernatant was discarded to eliminate blebs. Thereafter, apoptotic MØs were isolated with Annexin V-coated magnetic beads. Briefly, the cell pellet was suspended in binding buffer and incubated with Annexin V-coated magnetic beads. After 15 min, the cells were recovered by placing the column in a magnetic field and eluting the cells of interest with binding buffer. The efficiency of the purification procedure was monitored by flow cytometry and FITC/Annexin V staining. Apoptotic protein content was quantitated by Lowry´s method and generating a standard curve with bovine serum albumin (BSA).

### Intracellular trafficking of apoptogenic mycobacterial proteins within apoptotic MØs

To study the intracellular localization of apoptosis-inducing proteins by immunofluorescence, 5 x 10^5^ MØs were incubated with 50 μg CFSE-labeled MsmegLpqH cell wall proteins for 1 h. Thereafter, MØs were stained with TUNEL as described above and were mounted with an anti-fade reagent and DAPI. Cytospin slides were examined with a Zeiss LSM 5 Pascal laser-scanning confocal microscope equipped with a mercury lamp and fitted with Ar, HeNe 543 nm, and HeNe 633 nm lasers using the LSM5 Pascal 2.8 software Nuclei stained with TUNEL and/or nuclei containing translocated mycobacterial CFSE-labeled proteins were counted in at least 500 cells. To detect mycobacterial proteins present in the nuclei of MØs undergoing apoptosis, nuclei of 10 x 10^6^ MØs suspended in RPMI 1640 were incubated with 25 μl cytochalasin B (4.2 mM) for 30 min at 37°C. After a rinse in PBS, the cells were resuspended in nuclear buffer [10 mM HEPES (pH 7.4), 10 mM KCl, 2 mM MgCl_2_, 1 mM DTT, 2 μg/ml leupeptin, 2 μg/ml pepstatin, and 2 μg/ml aprotinin-containing cytochalasin B (10 µM)] and disrupted with a glass homogenizer. Released nuclei were passed through a 30% saccharose gradient (0.88 M) in nuclear buffer (500 ml) and separated in 15% PAGE-SDS gels. After the proteins were transferred to PVDF membranes, they were incubated with a rabbit antiserum to MsmegLpqH cell-wall proteins (diluted 1:200), or with a mAb recognizing LpqH (diluted 1:200), at 4°C overnight. After rinsing, the membranes were incubated with horseradish peroxidase (HRP)-conjugated secondary antibodies diluted 1:1000. After 1 h, reactive bands were visualized with a SuperSignal West Dura kit (Pierce, Rockford, IL, USA) or by DAB/H_2_O_2_ staining.

### Generation of DCs from bone marrow precursors

The method proposed by Inaba et al. was followed with minor modifications [19]. Briefly, bone marrow cells were obtained as described above and were cultured in RPMI 1640 supplemented with 15% FBS, 20 μg/ml gentamicin, 100 μl 0.1M 2-mercaptoethanol, and 25 ml/L 1M HEPES. To drive DC differentiation, 20 ng/ml of recombinant GM-CSF was added to the culture medium. After 3 d, complete culture medium was added. On day 6, immature DCs growing in conglomerates of non-adherent cells were recovered and rinsed at 453 x g for 5 min with PBS. The phenotype of the DCs was analyzed by flow cytometry with a mAb recognizing CD11c.

### Assay to determine the capacity of DCs to phagocytose apoptotic MØs

Since the uptake of antigenic material is a prerequisite for antigen processing and presentation to T cells [20], the capacity for immature DCs to phagocytose apoptotic cells was analyzed. Briefly, PKH-26-labeled MØs were induced to undergo apoptosis by exposure to cell wall proteins of MsmegLpqH or UV light. Purified apoptotic MØs (50 μg protein) were cocultured with immature DCs (5 x 10^5^ cells) labeled with PKH-67; 50 μg protein of apoptotic MØs correspond to 200,000 cells. To synchronize phagocytosis, apoptotic MØs were pelleted by centrifugation (1000 rpm, 5 min, 4°C) onto DC slides. After 1 h, 4 h, and 24 h, the cells were examined by flow cytometry and the percent of phagocytosis was determined. In addition, in assays with PKH-26 labeled apoptotic MØs, phagocytosis was analyzed setting a geometric mean fluorescence index (MFI). For fluorescence microscopy a sample of each set of cells was rinsed with PBS, fixed with 1% paraformaldehyde, and mounted with an anti-fade reagent and DAPI onto prepared cytospin slides. The cells were examined with an Olympus BX51 immunofluorescence microscope and with a LSM 5 Pascal Zeiss laser scanning confocal microscope with Software 2.8.

### Maturation phenotype of DCs cocultured with apoptotic MØs and cytokine release

Immature DCs were obtained from bone marrow precursor cells as described above. Briefly, 5 x 10^5^ DCs were incubated in RPMI 1640 supplemented with 5% FBS, for 24 h with 50 μg MsmegLpqH or UV-induced apoptotic MØs. As a positive activation control, DCs were treated with 1 μg of LPS. Each set of cells was stained with mAbs recognizing CD11c, antigen presentation molecules, MHC-I and MHC-II, and costimulatory molecules, CD40, CD80, and CD86. The CD11c^+^ cells were gated and a geometric mean fluorescence index (MFI) of the markers was set. Cytokine release in the supernatant of the DCs cocultured with apoptotic MØs for 24 h was also assayed. According to manufacturers’ instructions, a sandwich enzyme-linked immunosorbent assay (ELISA) method with mAbs recognizing IL-12, TNF-α, TGF-β, and IL-10 was used. The levels of cytokines released into the culture medium were calculated based on absorbance values measured at 450 nm with a microplate reader and standard curves that were generated with recombinant cytokines. The results are expressed in picograms per milliliter.

### Activation of T cells

Mononuclear spleen cell suspensions were obtained from naïve 6-week-old BALB/c mice. Briefly, spleens were resected, placed in Petri dishes containing RPMI 1640, and physically disrupted with a cell strainer. The resulting cell suspension was rinsed with RPMI 1640 and centrifuged for 5 min at 652 x g. To isolate T cells, erythrocyte lysis was performed. Then, the spleen mononuclear cells were labeled with a mAb to the pan-T cell marker Thy-1.2 (CD90.2) [21] that was diluted 1:1000 in PBS/FBS. After 30 min, the T cells were extensively rinsed and sorted with a FACSAria cell sorter (BD Pharmingen, Franklin, NJ, USA). The isolated T cells were labeled with 1 μM CFSE in serum-free PBS for 5 min at room temperature and then washed with RPMI 1640 supplemented with 10% FBS to remove unbound CFSE. The DCs that were activated with LPS or with apoptotic MØs were cocultured with CFSE-labeled T cells in flat-bottomed 96-well plates at 1:10 ratio. After 3 d, the cells were harvested and stained with anti-mouse CD4 and anti-mouse CD8 antibodies to assess cell proliferation by CFSE dilution. The cells were analyzed with flow cytometry and FlowJo version 7.6.2 software. The CD4^+^ and CD8^+^ populations were gated and the percentage of cells within each gate was recorded. We measured the cytokines released in the supernatant with an ELISA assay for IL-12, TNF-α, IL-10, and IFN-γ as described above. In addition, proliferation assays were performed with purified CD4 and CD8 T cells obtained from mice spleens by cell sorting. Purified cells were labeled with CFSE (1 μM) in serum-free PBS for 5 min at room temperature and cocultured for 3 days with DCs activated with MsmegLpqH or UV apoptotic MØs at a 10:1 ratio. Flow cytometry CFSE dilution assays were performed to assess cell proliferation.

### Cross-presentation assay

Cross presentation assays with purified CD4 and CD8 T cells were carried out. DCs were treated for 1 h with the proteasome inhibitor, MG-132 (0.2 μm), and with the proton pump inhibitor bafilomycin (0.05 μm) [22]. After 1 h, 5x10^5^ DCs were incubated with 50 μg MsmegLpqH or UV-induced apoptotic MØs protein for 24 h. After rinsing, the DCs were cocultured with CFSE-labeled, cell sorted CD8^+^ or CD4^+^ T cells at a 1:10 ratio. After 3 d, cell proliferation was assessed by CFSE dilution flow cytometry. Supernatants were collected and levels of IFN-γ were quantitated with a BioLegend ELISA kit.

### Statistical analysis

Statistical analyses were performed with GraphPad PRISM software (version 5.01; San Diego, CA, USA). Data expressed as the mean ± standard deviation were analyzed with non parametric Kruskal Wallis with Anova, Student´s t-test, Wilcoxon signed test and Mann Whitney test.

## Results

### Induction of MØ apoptosis with MsmegLpqH cell wall proteins

**Fig 1:**
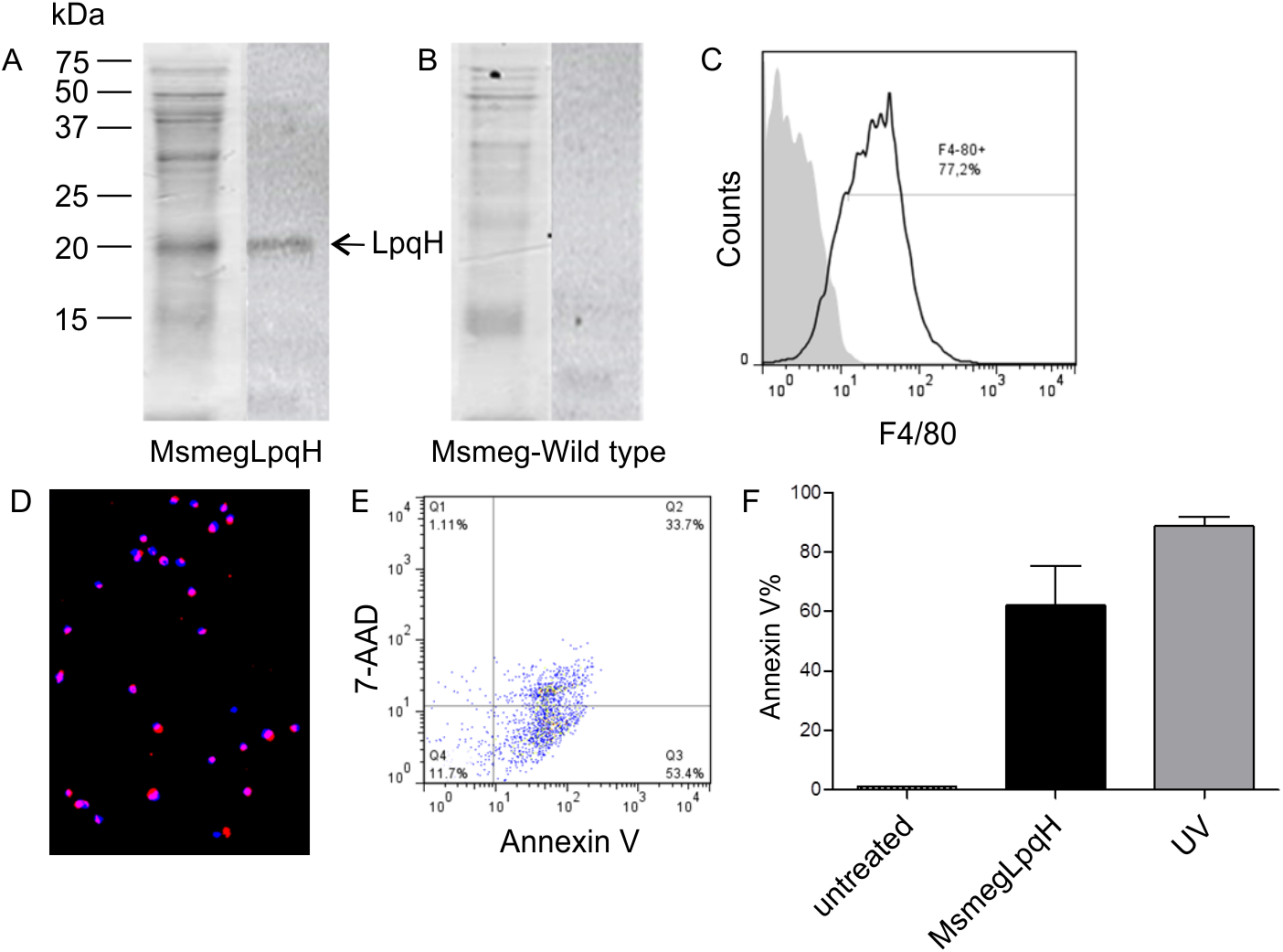
Mycobacterial cell wall proteins mediate apoptosis of bone marrow MØ. A *M*. *smegmatis* strain was transformed to overexpress LpqH, a 19 kDA Mtb cell wall lipoprotein that is apoptogenic for MØs. The cell wall proteins of the transformed strain were disrupted with sonication and then separated by 15% SDS-PAGE. (A) A Coomassie blue stained gel is shown and LpqH (19 kDa) was detected by immunoblot with a mAb and HRP labeled rabbit antiserum. (B) Immunoblot failed to reveal LpqH in wild-type *M*.*smegmatis*. (C) Flow cytometry revealed that the great majority of MØs used in these assays were F4/80 positive. (D, E) Following the incubation of bone marrow-derived MØs with 50 μg cell wall protein for 24 h, high levels of apoptosis were revealed by immunofluorescence microscopy of TUNEL assays (excitation 496 nm, emission 575 nm) (original magnification, 20x) and by flow cytometry with Annexin V. A representative Annexin/ 7-AAD dot plot showed that 87.1% of MØs were apoptotic and 33.7% were necrotic. (F) Treatment of MØs with UV light also induced high levels of apoptosis.

LpqH is a Mtb cell wall glycolipoprotein that stimulates T-cell mediated immune responses [23] and is highly apoptogenic for MØs [24]. Therefore, an *M*. *smegmatis* strain was transformed to express LpqH. The cell wall fraction from this strain was isolated by sonication and separated by SDS-PAGE. A subsequent immunoblot with a monoclonal antibody recognizing LpqH identified a 19-kDa band (Fig 1A) that was not present in wild-type *M*. *smegmatis* (Fig 1B). MØs differentiated from bone marrow precursors analyzed by flow cytometry were >75% positive for the F4/80 macrophage cell marker (Fig 1C). To induce apoptosis, MØs were incubated with the MsmegLpqH cell wall extract for 24 h. Both TUNEL and flow cytometry with FITC-labeled Annexin V to detect phosphatidylserine on the surface of cells detected high levels of apoptosis (Fig 1D and 1F). Flow cytometry using 7-ADD showed that 33.7% of the apoptotic MØs were also necrotic (Fig 1E). Also, treatment with UV light resulted in high apoptosis levels (Fig 1F). Next, apoptotic MØs were purified. Thereafter, the MØs were centrifuged at 453 x g to eliminate blebs in the supernatant, the MØs were purified by magnetic separation with Annexin V-coated microbeads. Flow cytometry analysis of the isolated MØs indicated up to 95% purity (data not shown).

### Translocation of mycobacterial antigens to the nuclei of apoptotic MØs

**Fig 2:**
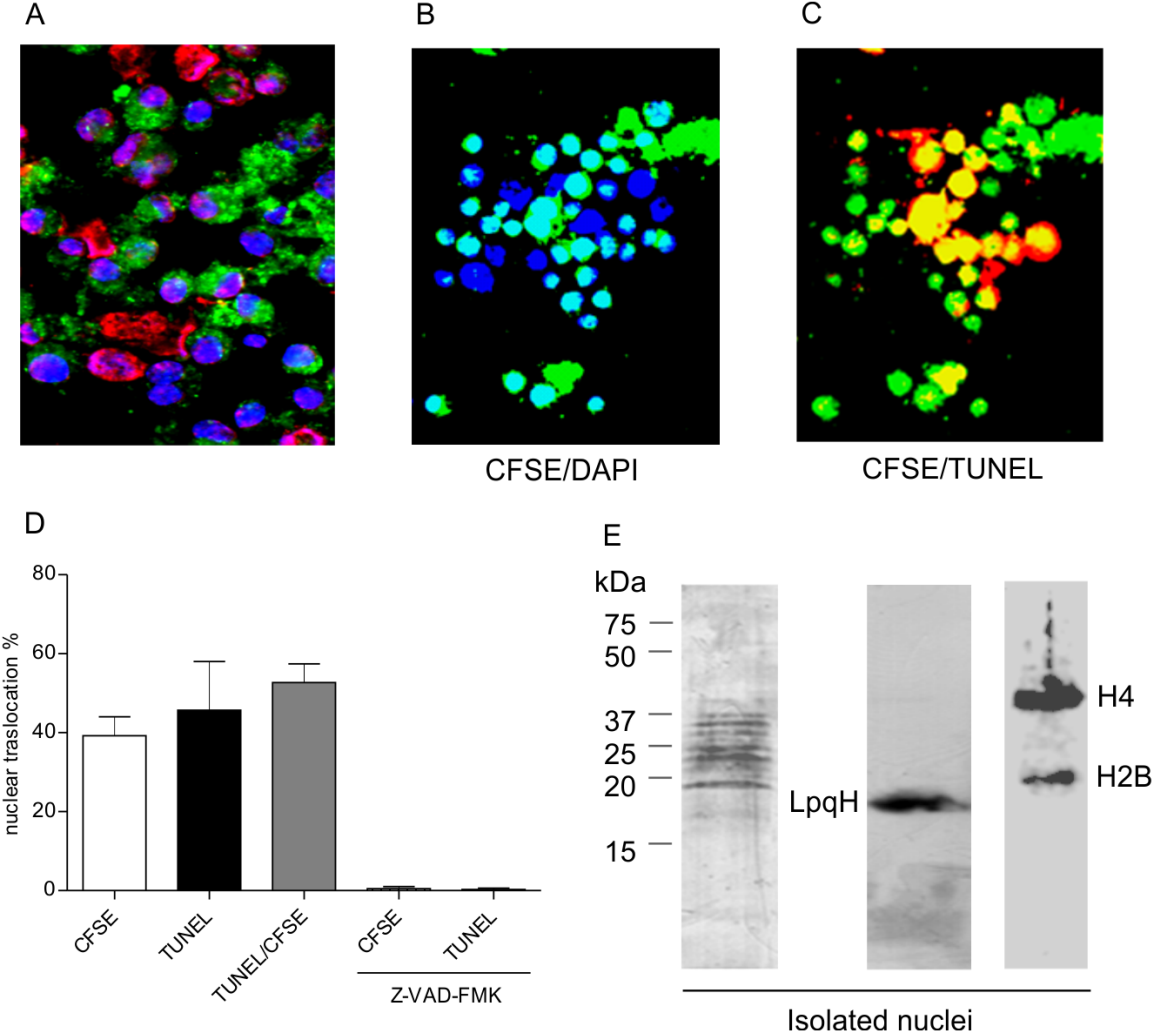
Mycobacterial antigens are translocated from the cytosol into the nuclei of apoptotic cells. To examine the intracellular movement of proteins that induce apoptosis, MØs were incubated with CFSE-labeled MsmegLpqH cell wall proteins. (A) After 1 h, MØ nuclei were stained with DAPI and TUNEL. Confocal immunofluorescence images show cytoplasmic vacuoles containing phagocytosed mycobacterial proteins in most of the cells (original magnification, 40x). (B, D) In addition, 39.9% of the cells exhibited overlapping DAPI (excitation 359 nm, emission 461 nm) and CFSE fluorescence (excitation 493 nm, emission 525 nm) (original magnification 40x). (C, D) Thus, translocation of mycobacterial proteins from cytosolic deposits to the nuclei was observed; 45.7% of the nuclei were apoptotic as shown by TUNEL, and 52.6% of the apoptotic nuclei exhibited overlapping TUNEL and CFSE fluorescence (original magnification 40x). (D) Nuclear translocation of proteins and apoptosis were virtually eliminated when the MØs were pretreated with the pancaspase inhibitor, Z-VAD-FMK. To identify the mycobacterial proteins that translocated to the nucleus, MØ were incubated with MsmegLpqH cell wall proteins for 1 h. (E) Nuclear extracts of these cells were then subjected to immunoblotting with a rabbit anti-*M smegmatis* antiserum and a secondary HRP labeled anti-rabbit IgG antibody. Several antigenic bands were observed that ranged in size from 20 kDa to 37 kDa. Staining with a mAb confirmed the nuclear translocation of LpqH and bands with sizes of 37 kDa and 20 kDa corresponding to histones H4 and H2B. The results shown are representative of three independent experiments.

To investigate the intracellular localization of mycobacterial proteins following their uptake by MØs, MsmegLpqH cell walls were labeled with CFSE and nuclei were stained with DAPI. In addition, DNA breaks in apoptotic nuclei were detected with TUNEL. One hour after phagocytosis, 400–800 cells were examined with confocal microscopy. CFSE-labeled granular material was observed in the cytoplasm of most of the MØs (Fig 2A). In addition, translocation of the CFSE-labeled apoptogenic proteins was observed in 39.2% of the nuclei based on the overlap of CFSE fluorescence and DAPI fluorescence (Fig 2B and 2D). In the TUNEL assay, 45.7% of the nuclei were apoptotic, with 52.6% of the apoptotic nuclei exhibiting an overlap of the TUNEL fluorescence with the CFSE-labeled mycobacterial proteins (Fig 2C and 2D). These findings suggest that the nuclear pores became abnormally permissive, thereby allowing mycobacterial proteins to pass from the cytosol into the nucleus. Accordingly, it has been reported that during apoptosis caspases can alter the structure of nuclear pore structure [25]. To test this possibility, we preincubated the MØs with the general caspase inhibitor Z-VAD-FMK. This treatment led to a marked decrease in both the translocation of CFSE-labeled proteins to the nuclei and the number of TUNEL positive nuclei (Fig 2D). To identify the mycobacterial proteins that translocated to the nuclei, MØs were incubated for 1 h with cell wall proteins obtained from MsmegLpqH. Following a subsequent extraction and SDS-PAGE of the nuclear proteins, immunoblotting with a rabbit anti-*M*. *smegmatis* antiserum and a secondary HRP labeled anti-rabbit IgG antibody revealed several antigenic bands with molecular weights ranging from 20–37 kDa (Fig 2E left). Thus, translocation of LpqH to the nuclei was demonstrated (Fig 2E center). In addition 37 kDa and 20 kDa bands corresponding to histones H4 and H2B were identified (Fig 2E right).

### Phagocytosis of apoptotic MØs by immature DCs

**Fig 3:**
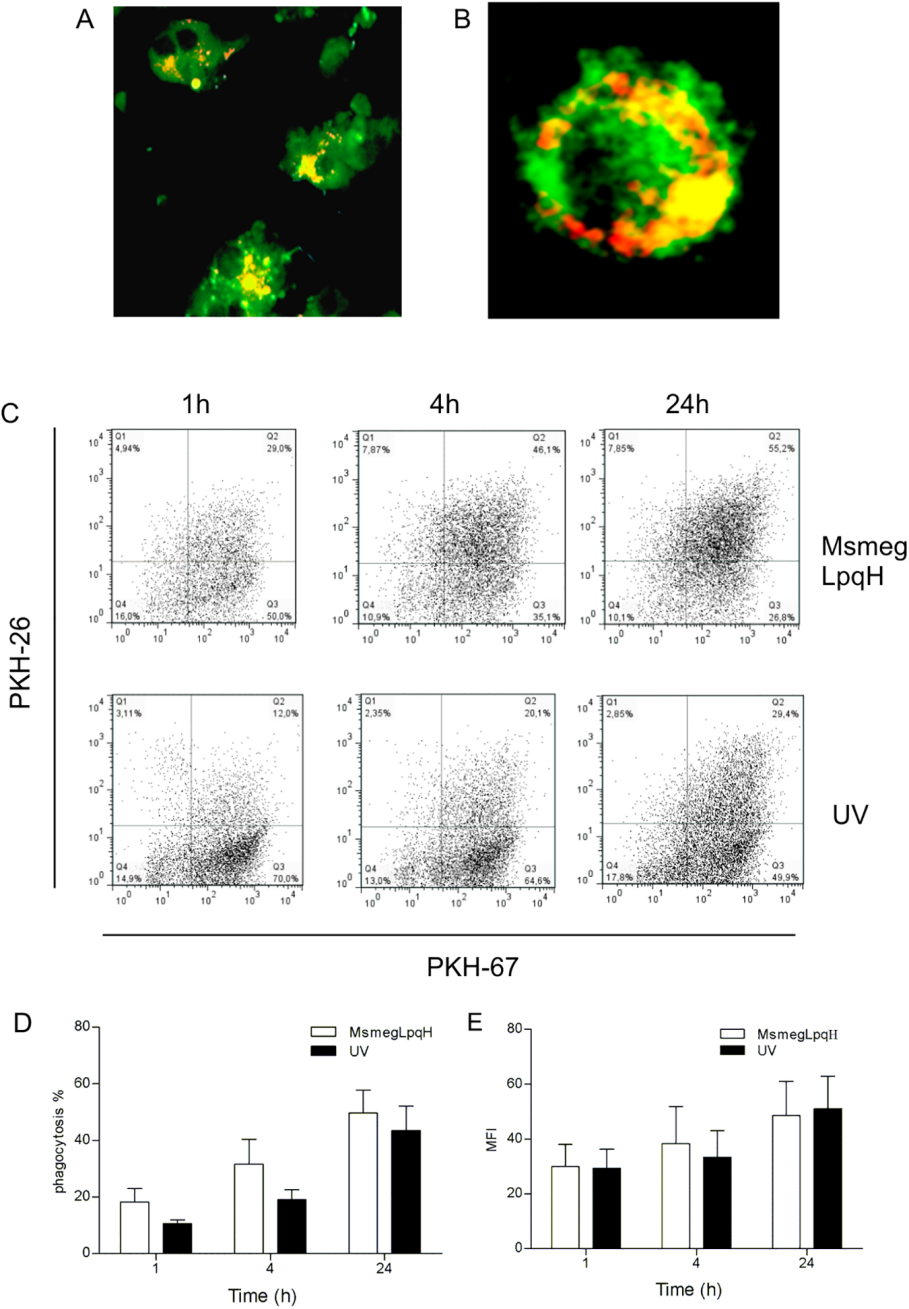
Inmature DCs efficiently phagocytose apoptotic MØ. DC precursors were obtained from mouse bone marrow and were cultured in the presence of GM-CSF for 6 days. Phagocytosis assays were then conducted with PKH-67-labeled DCs and apoptotic PKH-26-labeled MØs. (A) With confocal microscopy, classical DC morphology was observed (original magnification, 60x). (B) In addition, fluorescencent apoptotic bodies appeared to reside within vacuolar structures, (original magnification, 100x) (PKH-67 excitation 493 nm, emission 525 nm; PKH-26 excitation 496, emission 575 nm). (C, D) Flow cytometry of the DC/MØ cocultures at various time points showed that phagocytosis increased with time and high levels of phagocytosis were detected 24 h after coculturing. (C, D, E) Phagocytosis of UV light induced apoptotic bodies was similar. The results shown are representative of three independent experiments. MFI, Mean fluorescence index.

Immature DCs can phagocytose a variety of particles, including mycobacteria and apoptotic cells [26]. In this study, immature DCs that had undergone differentiation for 6 d in the presence of GM-CSF were labeled with PKH-67 and then incubated with MØs that were rendered apoptotic following their exposure to cell wall extracts from MsmegLpqH bacilli. The apoptotic MØs were isolated with Annexin V-coated magnetic beads and labeled with PKH-26 prior to their uptake. Confocal microscopy images of the mid-section of the DCs showed the presence of engulfed apoptotic bodies of various sizes (Fig 3A and 3B). Flow cytometry analysis further indicated that the phagocytosis was time-dependent, and 55.2% of the apoptotic material was taken up after 24 h (Fig 3C). High levels of phagocytosis were also observed for the cells that were exposed to UV light (Fig 3D and 3E).

### Maturation profile of the DCs that engulfed apoptotic MØ

**Fig 4:**
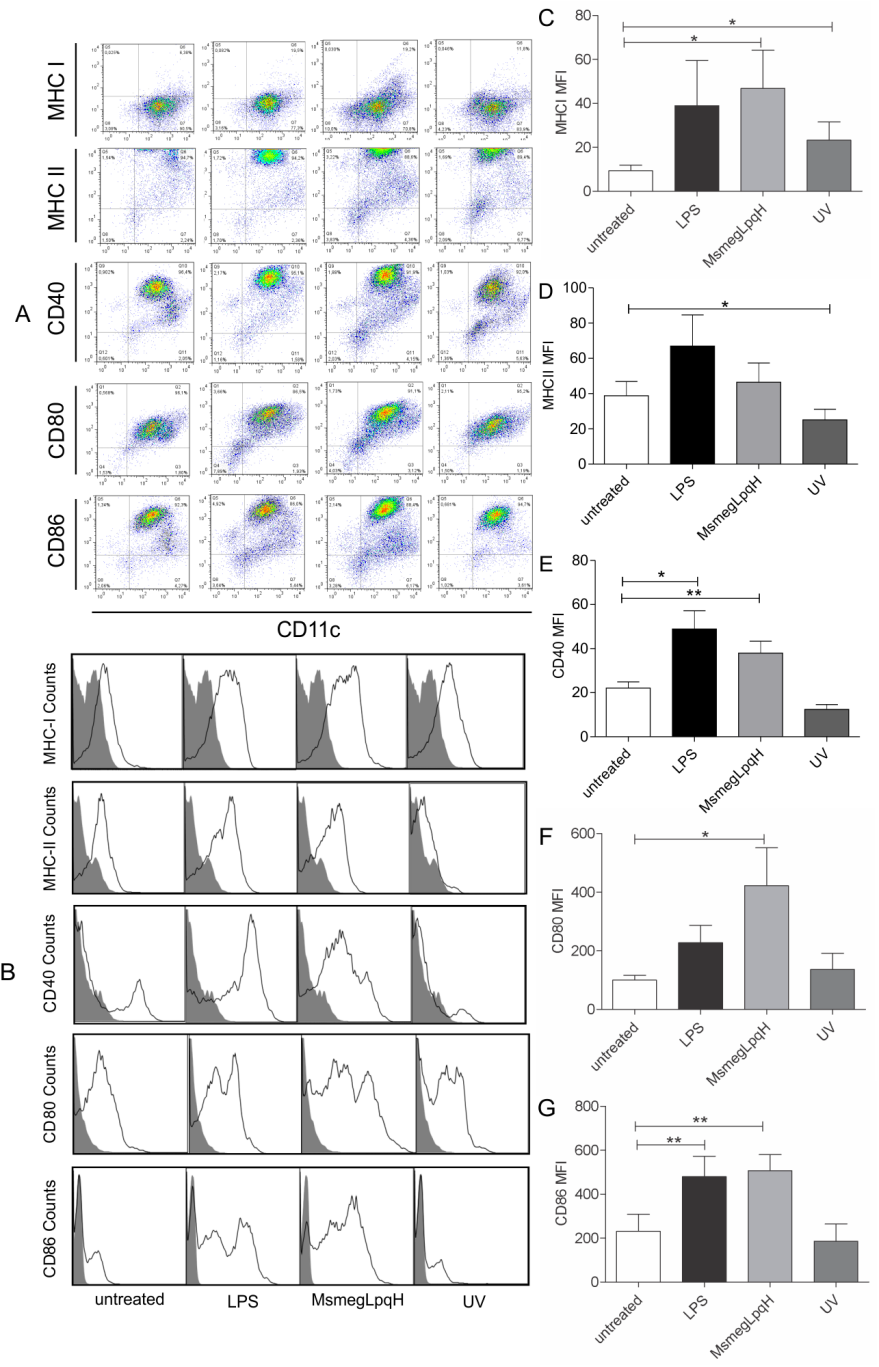
Phagocytosis of mycobacteria-induced apoptotic MØs induces an immunogenic profile for DCs. Immature DCs were obtained after 6 d of culturing bone marrow precursor cells and approximately 65–92% of this population was CD11c positive. These DCs were subsequently cocultured for 24 h with whole apoptotic MØ that were isolated with Annexin V-coated microbeads and were free of blebs. (A, B) Representative dot plots and histograms are shown. (C, D) In DC activated with MsmegLpqH apoptotic MØ the expression of MHC-I was upregulated (p ≤ 0.05; paired t Student’s test), while expression of MHC-II remained within basal values. (E, F, G) Expression of CD40 and CD86 were both greatly increased (p ≤ 0.05 and p ≤ 0.005, respectively; paired t Student’s test), while expression of CD80 increased to a lesser extent (p ≤ 0.05; Mann Whitney test). (C) DCs that engulfed UV apoptotic MØ exhibited upregulated MHC-I expression as well (p ≤ 0.05; Wilcoxon Signed rank test). (E, G) DCs activated with LPS increased the expression of CD40 (p ≤ 0.05; Mann Whitney test) and CD86 (p ≤ 0.002; paired t Student’s test). Results shown were obtained in five independent experiments.

To be immunogenic, DCs must mature and express a critical amount of antigen presentation and costimulatory molecules [20]. Here, DCs were grown for 6 d in the presence of GM-CSF. This treatment resulted in 65–92% of cells being CD11c positive and 20–40% of the cells expressing MHC-II. To eliminate blebs, after the induction of apoptosis, MØs were centrifuged at 453 x g and then isolated with Annexin V-coated microbeads. The DCs were then co-cultured for 24 h with bone marrow-derived MØs that were rendered apoptotic following exposure to MsmegLpqH cell walls or UV light. The markers were analyzed on the CD11c^+^ cells (Fig 4A). To analyze the expression of the maturation a MFI was set (Fig 4B). A significant increase in MHC-I expression was observed, and this increase was greater than that induced by LPS (Fig 4C). In contrast, levels of MHC-II remained within basal values (Fig 4D). Regarding costimulatory molecules, the DCs significantly overexpressed CD40, CD80, and CD86 (Fig 4E, 4F and 4G). The DCs that were challenged with UV apoptotic MØs overexpressed MHC-I, while the expression levels of MHCII and the costimulatory molecules detected were within basal values (Fig 4C, 4D, 4E, 4F and 4G). Activation with LPS increased the expression of CD40 and CD86.

### DCs cocultured with apoptotic bodies of MØ exhibit a mixed cytokine profile

**Fig 5:**
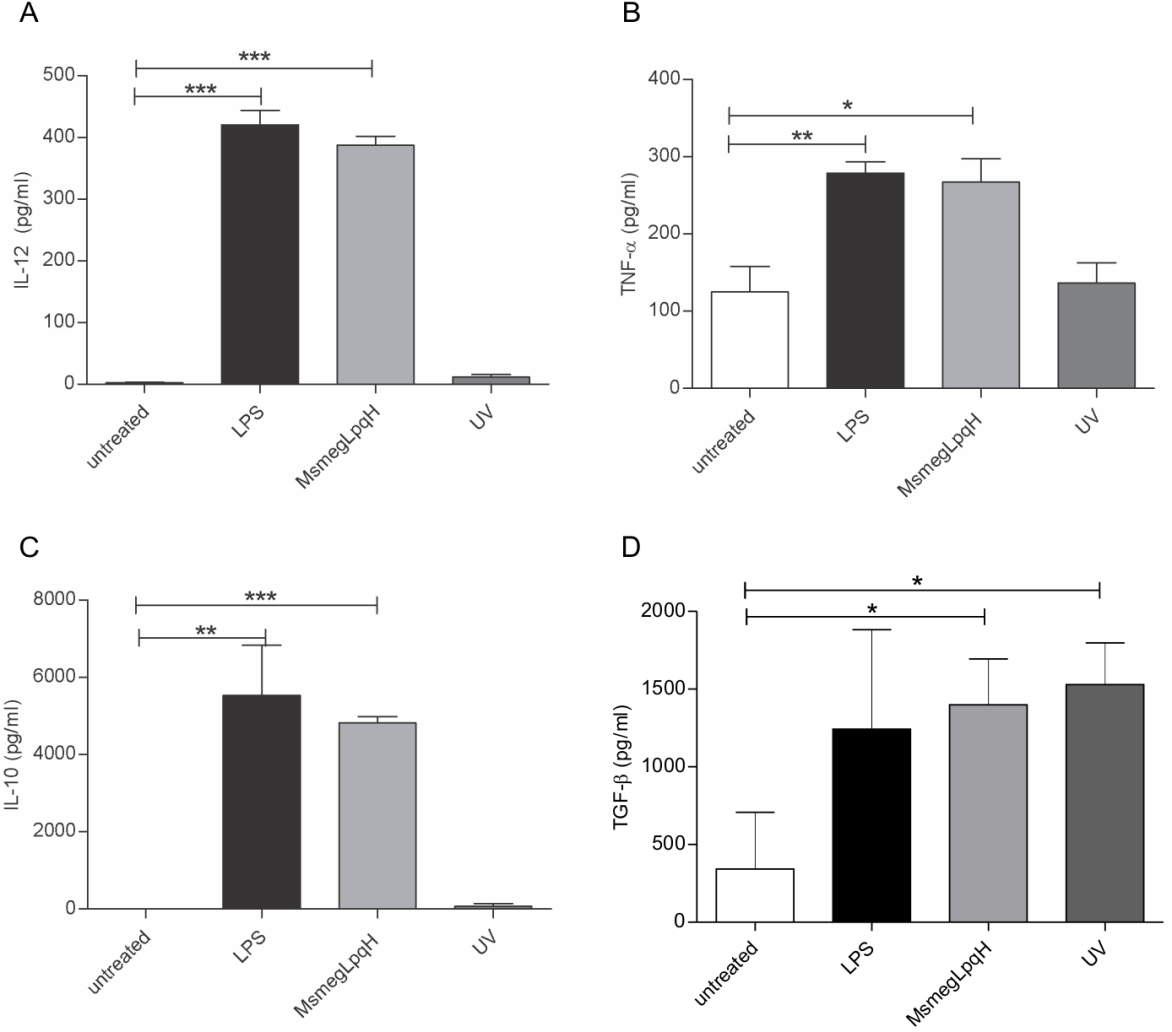
Phagocytosis of apoptotic MØ upregulates inflammatory and anti-inflammatory cytokines in DCs. When DCs (5 x 10^5^) were incubated for 24 h with MsmegLpqH-induced apoptotic MØs, (A, B, C, D) ELISAs of the coculture supernatants showed that secretion of IL-12 (p ≤ 0.0001) and TNF-α (p ≤ 0.007), as well as production of IL-10 (p ≤ 0.0001) and TGF-β (p ≤ 0.01), were increased. (D) In contrast, phagocytosis of the UV-treated MØs resulted in increased production of TGF-β (p ≤ 0.05), while the remaining cytokines detected were within basal levels. The results of four independent experiments are shown. For! L-12 and IL-10 analysis a paired t Student’s test was used. For TNF-α and TGF-β the Mann Whitney test was used.

The cytokines produced by activated DCs determine the type of T cell response that is elicited [27]. A capture ELISA method was used to quantify the cytokines present in the supernatant of the DCs that were challenged with MsmegLpqH-induced apoptotic MØs; increased secretion of IL-12 and TNF-α was observed (Fig 5A and 5B), and these two cytokines play a central role in antimycobacterial immunity [1]. Increased production of the anti-inflammatory cytokines, IL-10 and TGF-β, was also detected, and the level of IL-10 was 10-fold greater than the level of IL-12 (Fig 5C and 5D). In contrast, the cytokine release profile for the DCs that were incubated with the UV light-induced apoptotic MØ included basal levels of IL-12, TNF-α, and IL-10, and high levels of TGF-β.

### DCs activated with apoptotic MØs carrying mycobacterial antigens cross-present antigen to CD8^+^ T cells

**Fig 6:**
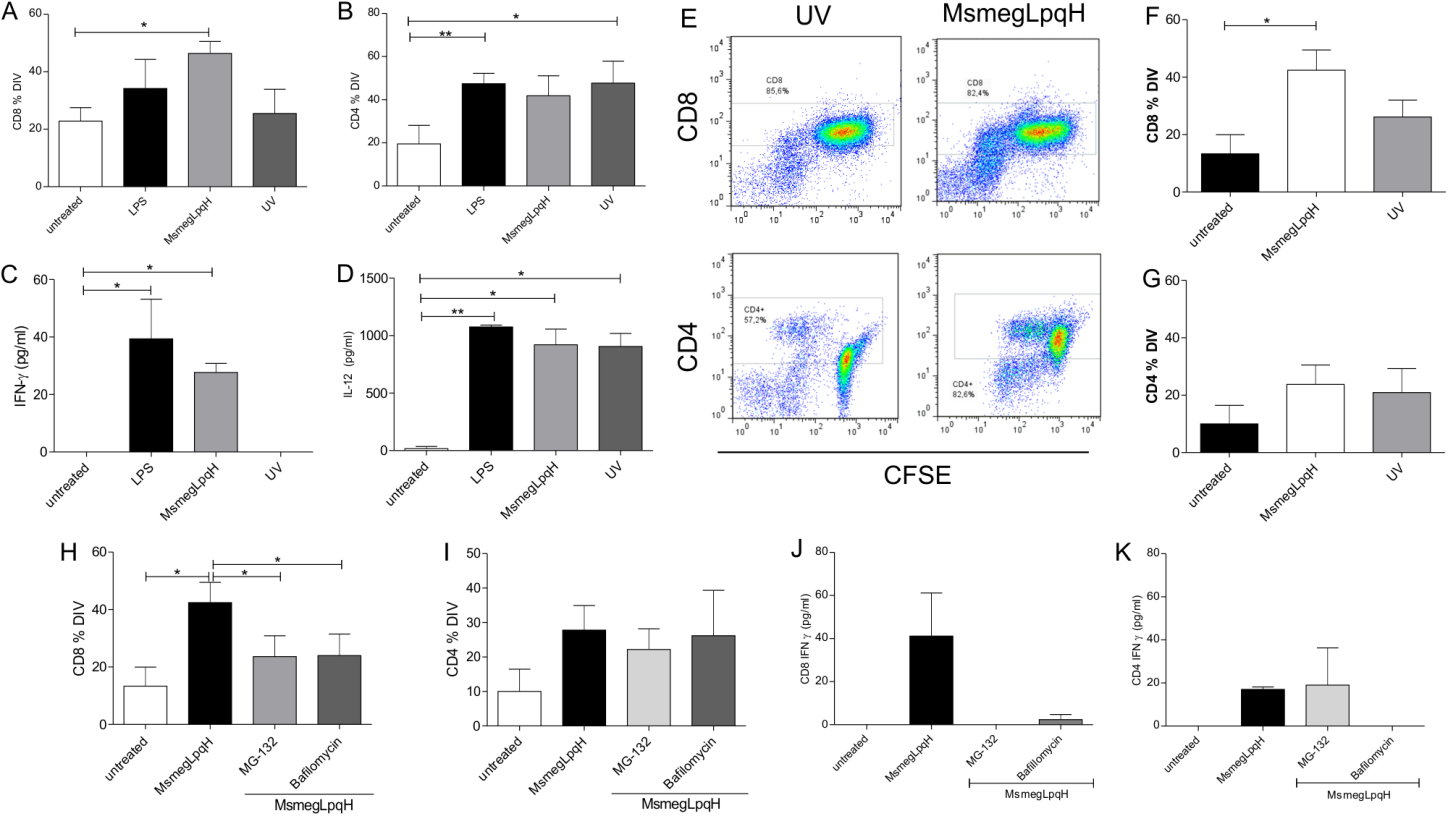
Induction of T cell proliferation by DCs activated with MsmegLpqH-induced apoptotic MØs. To examine the capacity of the DCs that engulfed the apoptotic MØs to activate T cells, DCs were cocultured with autologous T cells isolated from the spleens of naïve mice by cell sorting with an anti-Thy mAb. DCs and isolated T cells were cocultured at a 1:10 ratio for 3 d. T cell proliferation was assessed by the CFSE dilution method. (A, B) DCs that phagocytosed the mycobacteria-induced apoptotic MØs triggered the proliferation of CD8^+^ T cells, and not CD4^+^ T cells (p ≤ 0.04, paired t Student’s test). (C, D) In the supernatants increased amounts of IFN-γ (p ≤ 0.01; Kruskall-Wallis test) and IL-12 (p ≤ 0.01 Mann Whitney test). (B, C, D) In comparison, the DCs activated with UV-induced apoptotic MØs promoted the proliferation of CD4^+^ T cells and the release of IL-12 (p ≤ 0.001, Mann Whitney test) and IFN-γ was within basal values. (E, F, G) Proliferation assays with CD4^+^ and CD8^+^ T cells isolated by cell sorting confirmed the ability of CDs activated with MsmegLpqH apoptotic MØs to trigger a proliferative response of CD8^+^ T cells (p ≤ 0.05, paired t Student’s test) but not of CD4^+^ T cells. To identify the pathways used by DCs to activate CD8^+^ T cells, the DCs were treated with the proteasome inhibitor, MG-132, and a vacuolar H^+^ ATPase inhibitor, bafilomycin. (H) Both inhibitors reduced the proliferation of the CD8^+^ T cells (p ≤ 0.01 and p ≤ 0.02, respectively, Dunn’s Multiple Comparison test). (I) In comparison the proliferation of CD4+ T cells was not inhibited. The results of four independent experiments are shown. (J, K) Also, a representative experiment shows that both inhibitors decreased the production of INF-γ by the CD8^+^ T cells but not by CD4^+^ T cells.

The ability of DCs to generate T cell mediated immunity critically depends on the antigen presenting molecules involved. For example, presentation of antigen through MHC-II results in the activation of CD4 T cells, while presentation of antigen through MHC-I activates CD8 T cells [20]. To assess the ability of the DCs that engulfed apoptotic MØs to drive T cell activation, an autologous mixed lymphocyte reaction was performed with unfractionated spleen T cells that were obtained from naïve mice and were sorted with an anti-Thy antibody. It was observed that the DCs matured with MsmegLpqH apoptotic MØs activated the proliferation of CD8^+^ T cells (Fig 6A). Increased proliferation of CD4^+^ T cells was also observed, yet the increase was not statistically significant (Fig 6B). In comparison, both the DCs that were matured with LPS and the DCs that were incubated with MØs undergoing apoptosis induced by UV light triggered the proliferation of CD4^+^ T cells, and not the proliferation of CD8^+^ T cells (Fig 6A and 6B). The production of pro-inflammatory cytokines was subsequently quantitated in the supernatant with an ELISA kit. Secretion of IFN-γ and IL-12 were augmented in DCs activated with MsmegLpqH apoptotic MØs (Fig 6C and 6D). A similar observation was made for the DCs that were activated with LPS (Fig 6C and 6D). For the DCs that engulfed the UV-induced apoptotic MØs, their activation of T cells included increased levels of IL-12 and basal levels of IFN-γ (Fig 6C and 6D).

Following the above observations it was considered of interest to assess the ability of DCs to activate purified CD4^+^ and CD8^+^ T cells. Cells were cocultured with DCs as described before and the proliferation was measured (Fig 6E). It was observed that DCs activated with MsmegLpqH induced apoptotic MØ triggered significantly the proliferation of CD8^+^ T cells thus suggesting cross presentation of antigen (Fig 6F). The proliferative response of CD4^+^ T cells was mild and not statistically significant. The T cell response with DCs activated with UV apoptotic MØ was negligible (Fig 6G).

To confirm that the DCs that engulfed MsmegLpqH apoptotic MØs were cross-presenting antigen to CD8^+^ T cells, inhibition of cross-presentation was achieved by using naïve CD8^+^ T cells that were isolated from mouse spleens by cell sorting. The DCs were pretreated for 1 h with a proteasome inhibitor, MG-132 (0.2 μm), and with an inhibitor of vacuolar H^+^ ATPase, bafilomycin (0.05 μm). Similar treatments were used with purified CD4^+^ T cells. It was found that both MG-132 and bafilomycin similarly decreased the proliferation of CD8^+^ T cells (Fig 6H). In contrast the inhibitors had no significant effects on CD4^+^ T cell proliferation (Fig 6I). IFN-γ released by CD8^+^ and CD4^+^ T cells was measured in the supernatant. It was observed MG-132 and bafilomycin inhibited markedly the release of IFN-γ by CD8 T cells (Fig 6J). MG-132 had no effects on IFN-γ production by CD4^+^ T cells (Fig 6K).

## Discussion

Given the central role that DCs play in the adaptive T cell response [2], the aim of this work was to investigate the immunoregulatory effects of bone marrow-derived DCs that phagocytose apoptotic MØs. We found that DCs that engulfed apoptotic MØ carrying mycobacterial antigens developed a maturation profile that triggered the proliferation of CD8 T cells and IFN-γ production. There is growing evidence that supports the role of CD8 T cells in antimycobacterial immunity killing mycobacteria-infected host cells by secreting perforin, granzymes, and granulysin [28]. In addition, CD8 T cells produce IFN-γ and TNF-α which are important for mediating an immune response against mycobacteria [28].

During apoptosis, cells undergo dramatic changes [17]. In the early phases, dying cells release membrane bound microvesicles that are 100–1,000 nm in diameter (referred to as blebs) [29]. The cell that remains after blebbing constitutes an apoptotic body, and these are generally heterogeneous in size and contain most of the fragmented nucleus. It has been reported that blebs stimulate immunogenicity in DCs, whereas apoptotic cell bodies do not [30]. The changes that occur over time during apoptosis also appear to be important. For example, DCs that phagocytose cells in the early stages of apoptosis express an immature phenotype and their ability to activate T cells is low. In contrast, DCs that engulf cells that are in the late phase of apoptosis acquire an immunogenic profile [30, 31]. Until now, previous studies have only focused on the effects of apoptotic blebs that were released by mycobacteria infected MØs.

To activate DCs, we obtained late phase apoptotic MØs that were free of blebs. Apoptosis was induced by exposing MØ to extracts of mycobacterial cell wall proteins, particularly LpqH, an apoptogenic Mtb lipoprotein known to induce T cell immunity [23]. Intracellular trafficking of the apoptogenic proteins resulted in the translocation of these proteins from the cytosol to the nuclei of the cells undergoing apoptosis. Immunoblotting confirmed the presence of the translocated mycobacterial proteins into the nucleus, including LpqH. Furthermore, this phenomenon was virtually abolished in the cells that were pretreated with a caspase inhibitor. These findings are consistent with other studies that have shown that during apoptosis, caspases can alter nuclear pores, thereby allowing the passage of molecules [25]. The nuclear translocation of proteins in the context of mycobacteria-induced apoptosis has not previously been described. We hypothesize that the nuclear translocation of proteins may contribute to the immunogenicity of apoptotic cells.

It has been demonstrated that immature DCs endocytose apoptotic MØs with great efficiency [32]. In the present study, greater uptake of mycobacteria-induced apoptotic MØs was observed compared with the uptake of UV-induced apoptotic MØ. The maturation profile of DCs cocultured with MsmegLpqH apoptotic MØs included an increase in the expression of the costimulatory molecules, CD86 and CD40. The latter has a role in antimycobacterial immunity since it promotes the formation of MHC-peptide complexes and the production of IL-12 [27]. An important aspect of the DC maturation process is the production of cytokines that help shape T cell responses [33, 34]. In the present study, no distinct polarization pattern for autocrine cytokine production was observed, since both proinflammatory cytokines (IL-12, TNF-α) and anti-inflammatory cytokines (IL-10, TGF-β) were upregulated. However, the production of IL-10 was ten-fold higher than the production of IL-12. Similar findings were reported for DCs activated by LPS [33]. In addition, it was observed that IL-12 was initially released and then in the later stages IL-10 was released. IL-12 is a prototypic proinflammatory cytokine that is predominantly produced by activated DCs. It also plays a central role in the generation of Th1 responses [34]. In contrast, IL-10 inhibits the maturation of DCs, it down-regulates antigen presentation and costimulatory molecules, and it reduces the production of IL-12 and TNF-α [33, 35].

Following the phagocytosis of antigenic material, DCs process and present antigen to T cells via MHC-II, MHC-I, and CD1 antigen presentation molecules [20, 36]. In the present study, the phagocytosis of MsmegLpqH apoptotic MØs by DCs resulted in increased expression of MHC-I and an enhanced capacity to activate CD8 T cells. Previous studies have shown that DCs that have endocytosed whole apoptotic cells carrying exogenous antigens cross-present these antigens to CD8 T cells. For example, in a pioneer study, Albert et al. showed that the phagocytosis of apoptotic tumor cells infected with influenza virus by DCs resulted in the efficient cross-presentation of viral antigen to CD8 T cells [9]. In our study, treatment of DCs with either a proteasome inhibitor or a proton-pump ATPase inhibitor led to a decrease in CD8 T cell proliferation and IFN-γ release. These results indicate that proteasome and vacuolar acidification-dependent cross-presentation occurred. A requirement for endosomal acidification in proteasome-dependent cross-presentation has previously been recognized [22, 36, 37]. In addition, it was hypothesized that complex antigenic mixtures (e.g., apoptotic cells) could include proteolytic enzymes which could release antigens that would translocate to the cytosol and be processed by the proteasome [38].

## Conclusion

The results of the present study demonstrate that DCs that have phagocytosed MØs carrying mycobacterial antigens, acquire a mature phenotype which is characterized by increased expression of the antigen presentation molecule, MHC-I, and the costimulatory molecules, CD40 and CD86. Additionally, it was observed that the marked increase in production of the anti-inflammatory cytokine, IL-10, did not affect the ability of the DCs to cross-present antigen to CD8^+^ T cells. These effects may be due to the presence of mycobacterial antigens in the apoptotic bodies, since the DCs that engulfed the UV-induced apoptotic MØs were free of exogenous antigens and they exhibited a distinct phenotype that did not include upregulation of costimulatory and antigen presentation molecules or the production of proinflammatory cytokines. Furthermore, instead of activating CD8 T cells, the UV apoptotic bodies induced the proliferation of activated CD4 T cells.
